# Post-Mortem Examinations without Permission

**Published:** 1913-02-22

**Authors:** 


### NOTES ON LEGAL DECISIONS.

### Post-mortem Examinations without Permission.

There have been several instances of actions against doctors in respect of illegal or unauthorised *post-mortem* examinations, and in each of these there was put before the jury the simple issue whether the defendant wrongfully and illegally made such an examination whereby the plaintiff incurred damage. *Hughes* v. *Robertson*, 1912, 2 S.L.T., 505, was to a certain extent a case of that kind, but in addition it possessed quite a novel feature. Mrs. Hughes and her children brought the action against a Dr. Robertson, alleging that with ulterior motives, to which we shall refer later, he without authority, and in spite of explicit refusal, conducted a *post-mortem* examination upon the body of Thomas Hughes—the husband and father of the respective plaintiffs—who had met with an accident in the mine in which he worked, and subsequently died in the infirmary to which he had been removed.

So far, the case is comparatively simple, and it will be seen that if it were proved that the *post-mortem* was unauthorised, the plaintiffs would, according to Scotch law, be found entitled to damages in respect of injury to their feelings by such a harsh proceeding. Such damages, however, could not be very large in amount, hence the plaintiffs in their pleadings averred in addition special damage, and it is with regard to that item of their case that the decision is interesting. The deceased Hughes, on meeting with his accident, put in a claim for compensation against the mine-owners who employed him, and nearly a year after his death his representatives recovered same. Yet, it was in connection with this compensation claim that they sought to recover damages from the doctor. They alleged that Dr. Robertson was chief medical and surgical expert for the Coal Owners' Association of the mining district in which the accident took place, and in respect of his connection with the coal owners Dr. Robertson, they further alleged, had a strong interest to ascertain whether the inflammatory condition of Hughes' intestines —the immediate cause of his death, apparently— could be ascribed to any other origin than that of accident in the course of his employment, and to procure and preserve evidence to that effect. Accordingly, as a consequence of such interest, Dr. Robertson, it was averred, formed the desire and intention to procure a further examination of the body of Thomas Hughes, with the object of collecting and preserving evidence pointing to a cause of death other than accident in the mine, and ultimately effected his purpose without the authority, and in spite of the refusal, of the plaintiffs. In course of the *post-mortem* proceedings certain portions of the body of the said Thomas Hughes were, it was stated, removed by the defendant, in particular the whole affected portion of the intestines and the heart. These portions were not as they ought to have been replaced *in situ*, but, it was averred, were by the instructions, or at least with the assent and authority of defendant, taken away and burned or destroyed or otherwise dealt with for the purposes of the infirmary. In consequence of these alleged wrongous actings of the defendant, the plaintiffs stated that they had been made to endure an unnecessary amount of anxiety, delay, and expense in obtaining the compensation to which they felt themselves justly entitled. *Inter alia*, the solicitors for the deceased's trade society were authorised by its officials to inquire into the circumstances with a view to taking action on behalf of the relatives if such were justified, and for that purpose after ascertaining that a *post-mortem* had already been made to instruct a fresh one on behalf of the society. They did so, but in consequence of the previous mutilation of the body by the defendant the result was purely negative. In consequence, they felt themselves bound to advise the said society to withdraw their financial support, and the relatives were consequently at the necessity of finding fresh agents, and of instructing the prosecution of a claim on their own behalf. This, in their impoverished circumstances, they found it very difficult to effect, and in the result a very much longer time elapsed before they could establish their rights, and more expense was incurred than would have been necessary but for the alleged illegal actings of the defendant.

The case is instructive as showing how unexpected and varied in nature a damages action may be. In considering the pleadings, one gets the impression that the elaborate and detailed statements of the plaintiffs cover a thin case, so far as the actual damage sustained is concerned, but it is equally clear that they brought forward a competent and relevant action, for except when performed in virtue of the provisions of the Anatomy Acts, a *post-mortem* can only, failing consent, lawfully take place by permission of a competent legal authority, in England a coroner's warrant, in Scotland a sheriff's warrant. The Court recognised this and found that they must send the case to trial on the simple issue—whether the defendant did wrongfully make a *post-mortem* examination and dissection—and but for the novel averments regarding the compensation claim, the trial would have been before a jury. However, the nature and bearing of these statements on the preparation of a case under the Workmen's Compensation Act was so difficult and complicated a question that the Court sent the case to be tried before a judge. It need not be pointed out that this procedure gives a more favourable chance to the defendant of explaining satisfactorily the allegations made against him. Beyond that, it would, at this stage, be improper to say more, but the case, even at its present stage, merits notice in that it should tend to impress on practitioners the importance of not even seeming to act in the matter of *post-mortems* without authority deliberately asked and obtained.

